# Induced All-Female Autotriploidy in the Allotetraploids of *Carassius auratus* red var. (♀) × *Megalobrama amblycephala* (♂)

**DOI:** 10.1007/s10126-015-9647-7

**Published:** 2015-08-05

**Authors:** Qinbo Qin, Juan Wang, Jing Dai, YuDe Wang, Yun Liu, Shaojun Liu

**Affiliations:** Key Laboratory of Protein Chemistry and Developmental Biology of the State Education Ministry of China, College of Life Sciences, Hunan Normal University, Changsha, 410081 People’s Republic of China

**Keywords:** Distant hybridization, Gynogenesis, Autotriploid, Fertile, Unreduced gamete

## Abstract

Following activation by UV-irradiated BSB sperm, the fertilized eggs of tetraploid hybrids (abbreviated as 4nF_1_) (4*n* = 148, AABB) of *Carassius auratus* red var. (abbreviated as RCC) (2*n* = 100, AA) (♀) × *Megalobrama amblycephala* (abbreviated as BSB) (2*n* = 48, BB) (♂) developed into normal live gynogenetic offspring without chromosome doubling treatment. Some of these were autotriploids with three sets of red crucian carp chromosomes (abbreviated as G_1_) (3*n* = 150, AAA). G_1_ were all-females, and can produce unreduced (3n) eggs at age 1 year. After activation by UV-irradiated BSB sperm, the fertilized eggs of G_1_ developed into a second generation of autotriploid gynogenetic offspring (abbreviated as G_2_) (3*n* = 150) without chromosome doubling treatment. G_1_ were obviously different from both 4nF1 and RCC in their morphological traits and showed a significantly higher growth rate than RCC. In aquaculture, the autotriploid fish could provide an important source of gametes for the production of all-female triploid fish and for the establishment of autotriploid gynogenetic lines.

## Introduction

The introduction of triploid individuals into culture systems potentially minimizes the risks associated with accidental releases because triploids are usually sterile. In aquaculture, polyploids have been obtained by exposing fertilized eggs to physical or chemical agents (Teskered et al. 1993; Maclean et al. 1999; Linhart et al. 2001). Induction of triploid fish involves the retention of the second polar body of fertilized eggs, and tetraploid fish can be produced by suppression of the first mitotic division in fertilized eggs (Liu et al. 2007). Distant hybridization is an efficient way to produce crossbred fish that show heterosis with respect to growth rate and disease tolerance in culture (Hulata 1995; Hulata et al. 2008), and may generate tetraploid lines that can produce fast-growing sterile triploids by inter-ploidy hybridization (Liu et al. 2001, 2007, 2010; Liu 2010).

Usually, allopolyploids contain both parental genomes that undergo bivalent pairing at meiosis because only homologous chromosomes pair (Soltis and Soltis 2000; Wu et al. 2001). In face, abnormal chromosome behavior during meiosis has been observed among the polyploid hybrid progeny of many plants, which may lead to the production of unexpected gametes (Li and Heneen 1999).

In our previous study, we successfully obtained sterile triploid hybrids (3*n* = 124, AAB), fertile tetraploid hybrids (4*n* = 148, AABB), and fertile natural gynogenetic diploid (2*n* = 100, AA), but no diploid hybrids (2*n* = 74, AB) were found to survive in the first generation of *Carassius auratus* red var. (RCC, 2*n* = 100, AA) (♀) × *Megalobrama amblycephala* (BSB, 2*n* = 48, BB) (♂) (Liu et al. 2007; Liu 2010). The diploid hybrids probably did not survive because of the large difference in chromosome number between RCC (2*n* = 100) and BSB (2*n* = 48), presumably preventing the embryos from developing into viable fish. However, some diploid hybrid embryos developed into tetraploid hybrids through inhibition of the first cleavage which resulted in chromosome doubling (Liu et al. 2010).

Allotetraploids of RCC (♀) × BSB (♂) contain two sets of RCC-derived chromosomes and two sets of BSB-derived chromosomes, and would be supposed to produce allodiploid gamete (2*n* = 74, AB). However, abnormal chromosome behavior during meiosis in allotetraploids may lead to the formation of autotriploid gametes (3*n* = 150, AAA). There have been many reports on the fish gynogenesis using haploid eggs (Kavumpurath and Pandian 1994; Gomelsky et al. 1998; Hulata 2001), which have to be subjected to harmful treatment for doubling the chromosomes. The prominent advantage of the gynogenesis using autotriploid eggs is that it does not require the treatment for doubling the chromosomes. Thus, the autotriploids may be successfully obtained in gynogenetic progeny of allotetraploids. Interestingly, artificial autotriploids are usually sterile (Cherfas et al. 1994) but these autotriploids reached sexual maturity at age 1 year. This paper is the first to report of the formation of fertile autotriploid gynogenetic fish by distant hybridization and gynogenesis. These observations are of importance for both aquaculture and the genetic breeding of fish.

## Methods

### Animals and Crosses

BSB and RCC were obtained from the Protection Station of Polyploid Fish, Hunan Normal University. During the reproductive seasons (April) of 2004, 2005, and 2006, 4nF1 hybrids of RCC (♀) × BSB (♂) were produced. During the reproductive seasons of 2010, 2011, and 2012, gynogenetic offspring (G_1_) were obtained from 4nF1 eggs by artificial gynogenesis. During the reproductive season of 2011 and 2012, gynogenetic offspring (G_2_) were obtained from G_1_ eggs by artificial gynogenesis. The method of gynogenesis helps to clarify the ploidy of the eggs because polyploid eggs are able to develop into living fish but haploid eggs do not. The formation of G_1_ and G_2_ suggest that 4nF1 hybrids and G_1_ can produce the triploid egg.

### UV Irradiation for Sperm Inactivation

The milt was stripped from male BSB, diluted with Hank’s solution (1:4), and then poured into cold culture dishes to allow formation of a thin (0.1–0.2 mm) layer. UVC irradiation was performed using two quartz UV lamps (ZSZ20D) emitting a wavelength of 253.7 nm. The total UV dosage administered to the sperm over a period of 25–45 min was in the range 3000–3600 mJ/cm^2^. The milt suspension was continuously shaken at 30 rpm and 4 °C during the exposure period. The irradiation was monitored by observing the vitality of the sperm under a microscope. After UV irradiation, sperm were stored in glass tubes at 4 °C. All processes were conducted in the dark. This method enhanced the success rate of gynogenesis and decreased the number of hybrids in the offspring of gynogenesis.

### Preparation of Chromosome Spreads

Chromosome preparation is carried out on the kidney tissues of RCC, BSB, 4nF1, G_1_, and G_2_, according to the procedures reported by Liu et al. (2001, 2007). For each type of fish, 200 metaphase spreads (20 metaphase spreads from each of 10 fish) of chromosomes were counted and analyzed for further determination of ploidy and chromosome number. Examining the chromosomal spreads can directly identify the chromosomal number of RCC, BSB, 4nF1, G_1_ and G_2_.

### Gonadal Structure

Ten RCC, 10 4nF1, and 40 G_1_ at age 9 months were randomly selected for histological observation of gonad structure. Gonads were fixed in Bouin’s solution, embedded in paraffin, sectioned with a Leica RM2016 microtome, and stained with hematoxylin and eosin. Tissue sections were observed and photographed with an Olympus CX41 microscope. Gonadal development was staged according to criteria established for cyprinid fish (Liu 1993). Observation of gonadal development was used to determine whether the gynogenetic progenies were fertile.

### Morphological Traits

At 1 year of age, 20 RCC, 20 4nF1, and 20 G_1_ were randomly selected for morphological examination with reference to the standards set by Zou et al. (2008). Measurable traits recorded were the mean values of the whole length, the body length (excluding tail) and width, the head length and width, and the tail length and width. These values were used to calculate ratios of whole length to body length (WL/BL), body length to body width (BL/BW), body length to head length (BL/HL), head length to head width (HL/HW), tail length to tail width (TL/TW), and body width to head width (BW/HW) are calculated. Countable traits recorded for each fish were the number of: lateral line scales; scales above and below the lateral line; dorsal fin rays; abdominal fin rays; and anal fin rays. ANOVA and pairwise comparisons among the different types of fish were analyzed using SPSS Statistics 17.0 and used to identify similarities and significant differences (*P* < 0.01) between the gynogenetic progenies and their parents. Comparing the measurable and countable traits between the gynogenesis progenies and their parents is useful to identify the similarities and differences between the gynogenesis progenies and their parents.

### Fluorescence In Situ Hybridization

Species-specific centromere probe of fluorescence in situ hybridization (FISH) was made from RCC and amplified by PCR using the primers 5′-TTCGAAAAGAGAGAATAATCTA-3′ and 5′-AACTCGTCTAAA CCCGAACTA-3′. The FISH probes were produced by Dig-11-dUTP labeling (using a nick translation kit; Roche, Germany) of purified PCR products. FISH was performed according to the method described by He et al. (2012). For each type of fish (RCC, 4nF1, G_1_ and G_2_), 200 metaphase spreads of chromosomes from ten individuals are analyzed under an inverted microscope (CW4000, Leica, Germany) with a confocal imaging system (LCS SP2, Leica). Captured images were colored and superimposed in Adobe Photoshop CS6. FISH was used to identify the origin of the chromosomes in the gynogenetic offspring at the molecular level.

### Evaluation on the Aquacultural Performance of G_1_ and G_2_

Aquaculture data were collected in 2012 from three sites in Hunan: Wangcheng, Changsha, and Changde.

RCC, G_1_, and G_2_ fry were transported to their destinations 3 days after hatching and kept in separate, aerated aquaculture net cages at 21–25 °C for 1 week. They were fed two boiled egg yolks per 40–60 thousand fry. On the first and the last day, the net cage was thoroughly stirred, and then three 100-ml water samples were randomly taken from each net cage, combined and diluted to 2000 ml. Three 10-ml water samples were then taken from the 2000-ml sample and the numbers of fry in them were counted to estimate the mean density. The total number of fry was calculated from the mean density and the total volume of water in each cage. The survival rate was calculated based on the total number of fry on the first and last days.

On day 11 post-hatching, RCC, G_1_, and G_2_ were transferred to different ponds at a density of 120,000 fry/667 m^2^ at each site. They were fed with plankton and 3–5 kg of soybean milk daily. Twenty-one days later, subsamples of these fish were randomly selected and stocked at a density of 1800 fish/667 m^2^ for the adult grow-out stage. These were fed with aquatic compound feed containing 32 % protein. Three ponds were prepared for each fish taxon at each site. All fish were bred under similar conditions. The number of dead fish in each pond was recorded to calculate the survival rate during the adult grow-out stage from day 32 to day 217 post-hatching. The body weights of 30 randomly selected individuals of each taxon at each site were measured monthly from June to December. As there were no significant differences among the three sites in the mean survival rates and body weights of fish of the same type, the data were pooled for comparison of growth performance among RCC, G_1_, and G_2_. This method was used to determine whether the autotriploids (G_1_ and G_2_) grew more rapidly than RCC.

## Results

### Formation of Experimental Fish

**Fig. 1:**
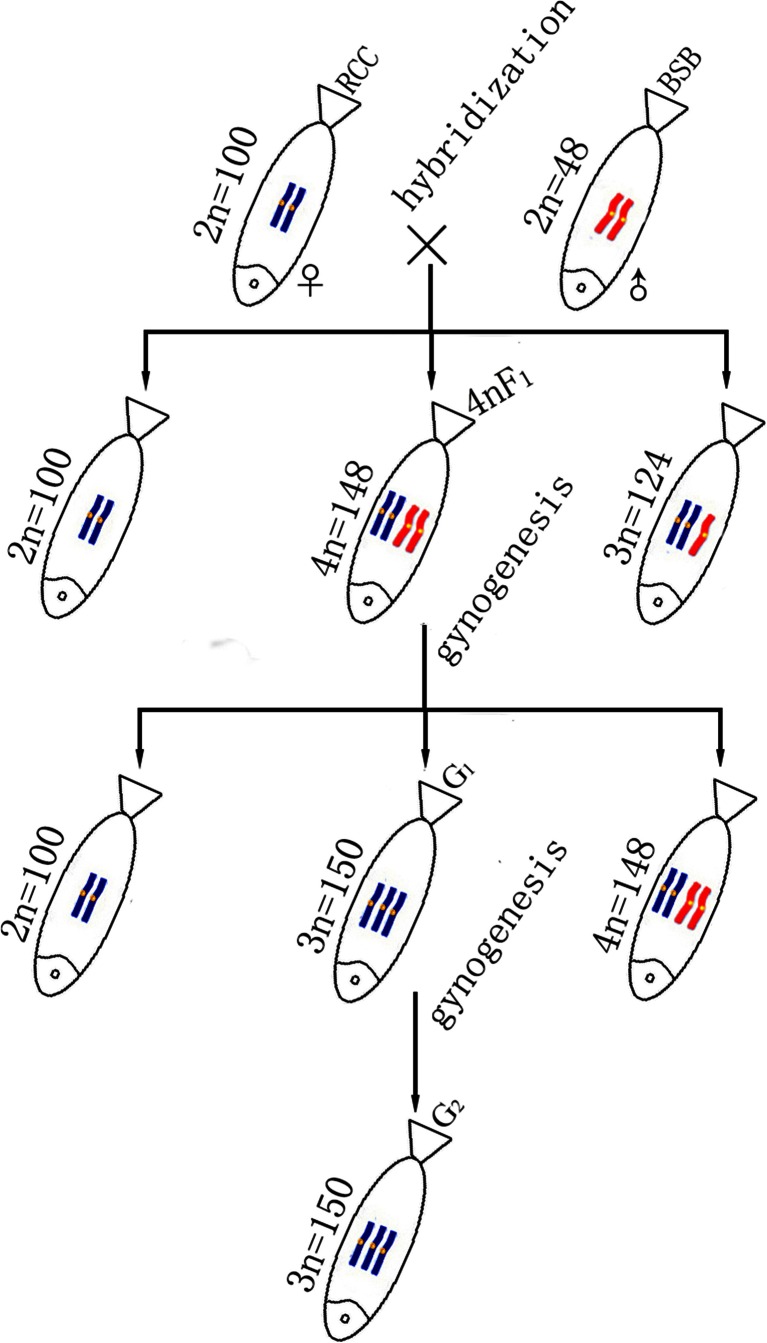
Crossing procedure used in the formation of the experimental fish. The parental origins of the chromosomes are indicated by *blue* and *red* colors

Following activation by UV-irradiated BSB sperm, the fertilized eggs of 4nF1 developed into viable gynogenetic offspring of normal appearance without chromosome doubling treatment. Autotriploids gynogenetic offspring (G_1_) accounted for 37.5 % of the total. Likewise, the fertilized eggs of G_1_ developed into second-generation gynogenetic offspring (G_2_) without chromosome doubling treatment after activation by UV-irradiated BSB sperm (Fig. 1). Autotriploids gynogenetic offspring accounted for 100 % of the total. The fertilization rates, hatching rates, and survival rates of the gynogenetic progeny of 4nF1 and G_1_ are shown in Table 1.

**Table 1:** The fertilization rates, hatching rates, and surviving rate in gynogenetic progeny of 4nF1 and G_1_

Egg source	Sperm source	UV-irradiated (min)	Fertilization rate (%)	Hatching rate (%)	Surviving rate (%)
4nF1	BSB	0	67.14 ± 5.21	56.45 ± 4.12	46.34 ± 3.14
4nF1	BSB	28	35.22 ± 3.52	28.56 ± 1.23	20.56 ± 1.24
4nF1	BSB	40	18.87 ± 3.21	11.65 ± 2.67	9.65 ± 2.65
4nF1	BSB	45	8.72 ± 2.32	5.29 ± 1.25	4.12 ± 1.48
G_1_	BSB	28	19.36 ± 3.21	11.63 ± 2.73	9.53 ± 2.19
G_1_	BSB	40	42.57 ± 2.92	38.65 ± 4.91	33.65 ± 2.23
G_1_	BSB	45	35.18 ± 6.21	28.89 ± 5.31	24.89 ± 3.54

Fertility rate = (number of fertilized eggs/number of eggs) × 100 %; Hatching rate = (number of hatching fry/number of eggs) × 100 %; Survival rate = (number of normal fry/number of eggs) × 100 %

### Examination of Chromosome Number

**Fig. 2:**
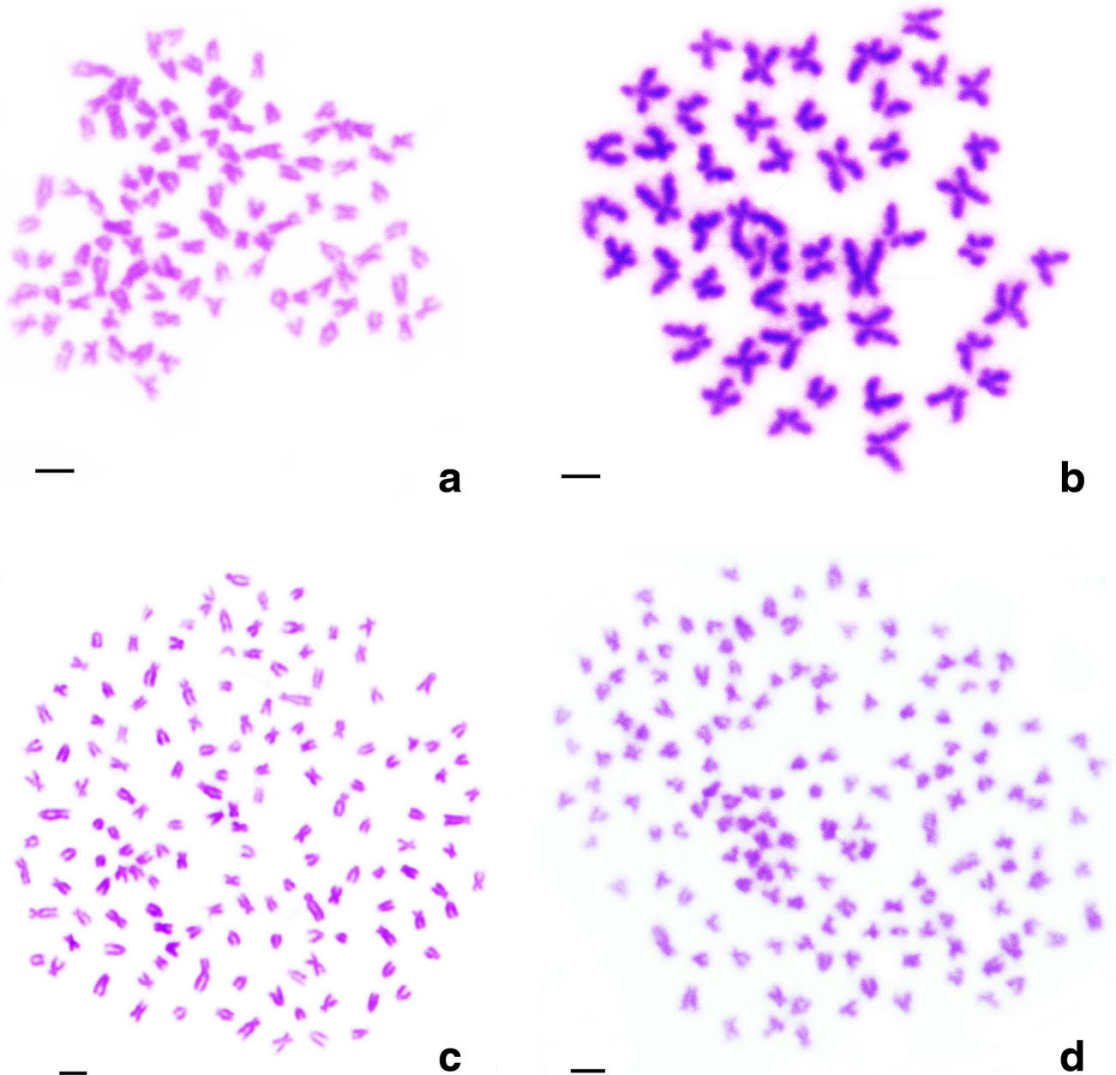
Chromosome spreads at metaphase in RCC, BSB, 4nF1, and G_1_. **a** The 100 chromosomes of RCC; **b** The 48 chromosomes of BSB; **c** The 148 chromosomes of 4nF1; **d** The 150 chromosomes of G_1_; *Bar* in **a**–**d**, 3 μm

Table 1 presents the distributions of chromosome numbers in RCC, BSB, 4nF1, G_1_, and G_2_. For RCC, 93 % of chromosomal metaphases had 100 chromosomes (Fig. 2a; Table 2). For BSB, 86 % of chromosomal metaphases possessed 48 chromosomes (Fig. 2b; Table 2). For 4nF1, 83 % of chromosomal metaphases had 148 chromosomes (Fig. 2c; Table 2). For G_1_, 81 % of chromosomal metaphases had 150 chromosomes (Fig. 2d; Table 2). For G_2_, 84.5 % of chromosomal metaphases have 150 chromosomes (Table 2).

**Table 2:** Examination of chromosome number in RCC, BSB, 4nF1, G_1_, and G_2_

Fish type	No. of metaphase	Distribution of chromosome number							
		<48	48	<100	100	<148	148	<150	150
RCC	200			14	186				
BSB	200	28	172						
4nF1	200					34	166		
G_1_	200							38	162
G_2_	200							31	169

### Fluorescence In Situ Hybridization

**Fig. 3:**
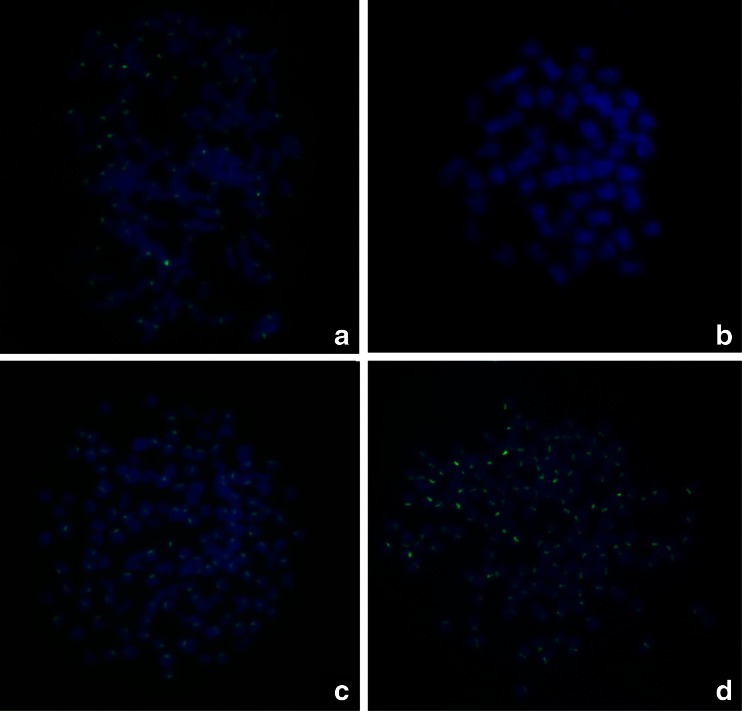
Examination of the FISH signals in RCC, BSB, 4nF1 and G_1_. **a** The centromere probe hybridized to 100 chromosomes in RCC; **b** No chromosomes of BSB hybridized; **c** The centromere probe hybridized to 100 RCC-derived chromosomes in 4nF1; **d** The centromere probe hybridized to 150 chromosomes of G_1_

The species-specific centromere probe (GenBank accession No.: JQ086761) hybridized with 100 chromosomes of RCC (Fig. 3a; Table 3), whereas no chromosomes of BSB showed hybridization (Fig. 3b; Table 3). Therefore, RCC and BSB-derived chromosomes could be discriminated by FISH using the centromere probes. As expected, the centromere probe also hybridized with 100 chromosomes of 4nF1 (Fig. 3c; Table 3), indicating that they possessed two sets of RCC-derived chromosomes. The centromere probe hybridized with 150 RCC-derived chromosomes in G_1_ and G_2_ (Fig. 3d; Table 3), indicating that they possessed three sets of RCC-derived chromosomes_._

**Table 3:** Examination of hybridizing signals by FISH in RCC, BSB, 4nF1, G_1_, and G_2_

Fish type	No. of fish	No. of metaphase	No. of loci
RCC	10	200	100
BSB	10	200	0
4nF1	10	200	100
G_1_	10	200	150
G_2_	10	200	150

### Morphological Traits

**Fig. 4:**
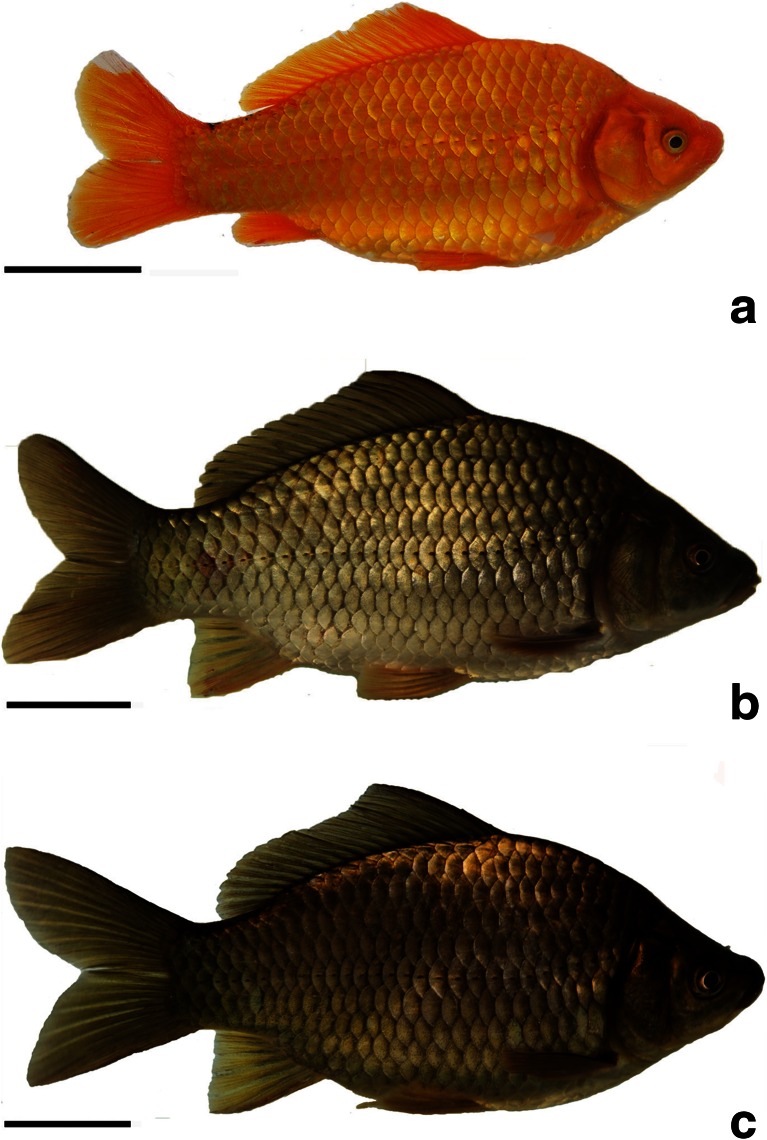
Morphological appearances of RCC, 4nF1, and G_1_. **a** RCC; **b** 4nF1; **c** G_1_; *Bar* in **a**–**c**, 4 cm

There were clear morphological differences among RCC (Fig. 4a), 4nF1 (Fig. 4b) and G_1_ (Fig. 4c). The body color of RCC was red, while that of 4nF1 and G_1_ are green-brown. An interesting difference was the presence of barbels in 4nF1 and their absence in G_1_. Tables 4 and 5 show the values for the measurable and countable traits in RCC, 4nF1 and G_1_. The ratios of the measurable traits all differed significantly between RCC and G_1_ (*P* < 0.01), except for HL/HW and BW/HW. The ratios BL/BW and BW/HW were significantly different between G_1_ and 4nF1. The ratios of all measurable traits differed significantly between 4nF1 and RCC (*P* < 0.01), with the exception of BL/BW and HL/HW.

**Table 4:** Comparison of ratios of the measurable traits between RCC, 4nF1 and G_1_

Fish type	WL/BL	BL/BW	BL/HL	HL/HW	TL/TW	BW/HW
RCC	1.22 ± 0.02^a^	2.18 ± 0.02^a^	3.72 ± 0.03^a^	1.07 ± 0.03^a^	0.82 ± 0.03^a^	1.84 ± 0.03^a^
4nF1	1.18 ± 0.02^b^	2.18 ± 0.02^a^	3.83 ± 0.03^b^	1.08 ± 0.04^a^	0.75 ± 0.04^b^	1.92 ± 0.02^b^
G_1_	1.19 ± 0.03^b^	2.21 ± 0.02^b^	3.82 ± 0.02^b^	1.08 ± 0.01^a^	0.75 ± 0.02^b^	1.85 ± 0.02^a^

The same superscript letters in the same column indicate no significant difference (*P* > 0.01); different superscript letters in the same column indicate significant difference (*P* < 0.01); mean value ± standard deviation

**Table 5:** Comparison of the countable traits between RCC, 4nF1 and G_1_

Fish type	No. lateral line scales	No. of scales above the lateral line	No. of scales below the lateral line	No. of dorsal fins rays	No. of abdominal fins rays	No. of anal fins rays
RCC	29.20 ± 0.70^a^ (28–30)	5.60 ± 0.50^a^ (5–6)	5.70 ± 0.47^a^ (5–6)	III + 18.65 ± 0.49^a^ (18–19)	8.55 ± 0.51^a^ (8–9)	III + 5.65 ± 0.49^a^ (5–6)
4nF1	31.65 ± 0.49^b^ (31–32)	6.55 ± 0.51^b^ (6–7)	6.45 ± 0.51^b^ (6–7)	III + 18.70 ± 0.98^a^ (17–20)	8.60 ± 0.50^a^ (8–9)	III + 6.40 ± 0.68^b^ (5–7)
G_1_	31.34 ± 0.43^b^ (31–32)	5.75 ± 0.23^a^ (5–6)	5.73 ± 0.27^a^ (5–6)	III + 18.2 ± 0.87^a^ (17–19)	9.12 ± 0.26^b^ (9–10)	III + 6.57 ± 0.36^b^ (6–7)

The same superscript letters in the same column indicate no significant difference (*P* > 0.01); different superscript letters in the same column indicate significant difference (*P* < 0.01); Ш represents hard fin rays and Arabic numerals represent soft fin ray; mean value ± standard deviation; the numbers in brackets indicate range for the value

All countable traits differed significantly between G_1_ and RCC (*P* < 0.01), with the exception of the number of scales above the lateral line, lower scales below the lateral line and dorsal fins rays. However, the numbers of scales above the lateral line, lower scales below the lateral line and abdominal fins rays differed significantly between G_1_ and 4nF1 (*P* < 0.01). With the exception of the numbers of dorsal fins and abdominal fins, all other countable traits differed significantly between 4nF1 and RCC (*P* < 0.01).

### Analysis of Gonadal Development

RCC, G_1_, and G_2_ reached sexual maturity at age 1 year, but 1-year-old 4nF1 did not produce mature eggs or sperm. Gonad development was observed in 40 G_1_, and showed that 90 % G_1_ possessed normal ovary structure and 10 % G_1_ possess abnormal. Testicular tissues were not found.

**Fig. 5:**
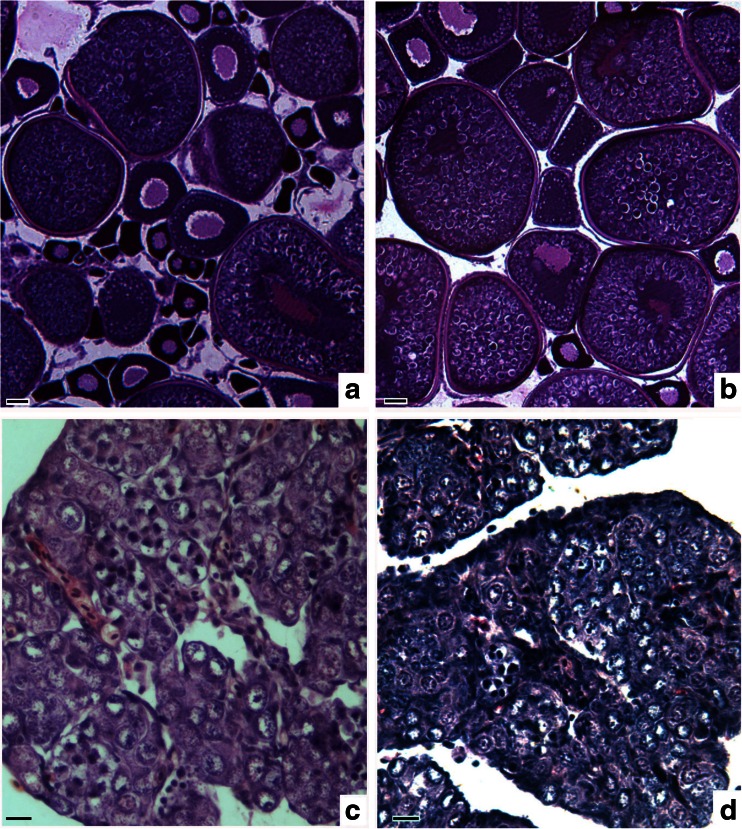
Gonadal microstructure in RCC, 4nF1, and G_1_. **a** The ovary of RCC, showing normally developed oocytes in phase III; **b** The ovary of G_1_, showing normally developed oocytes in phase III; **c** The ovary of 4nF1 is occupied by many oogonia; **d** Abnormal ovary of G_1_, showing proliferating oogonia that did not develop into oocytes. *Bar* in **a**–**d**, 50 μm

Figure 5 shows the microstructure of the ovaries RCC, 4nF1 and G_1_. The ovaries of RCC and G_1_ at 9 months old were normally developed and are mainly contained oocytes in phase III (Fig. 5a, b). However, at 9 months old, the ovaries of 4nF1 were occupied by many oogonia and very few oocytes (Fig. 5c). Oogonia proliferated in the abnormal ovaries of G_1_ but did not develop into oocytes, i.e., gonadal development was delayed (Fig. 5d)

### Aquaculture Performance

**Fig. 6:**
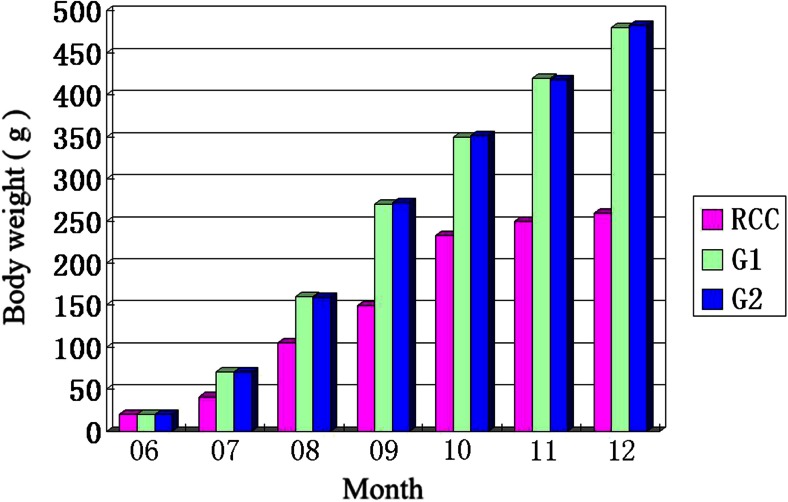
Growth of RCC, G_1_, and G_2_ from June to December

The survival rates of RCC, G_1_, and G_2_ ranged from 80.1 to 83.5 % during the fry stage and from 85.7 to 87.2 % during the adult grow-out stage; there were no significant differences among the taxa (*P* > 0.01) (Table 6). The mean body weight of RCC in December was 260 g, which was significantly smaller than that of G_1_ (480 g), and of G_2_ (482 g) (*P* < 0.01). Figure 6 presents the growth rates of RCC, G_1_, and G_2_ from June to December 2012. The growth rates of G_1_ and G_2_ were higher, and obviously increased, compared with those of RCC over the entire period.

**Table 6:** Comparison of growth performance between RCC, G_1_, and G_2_

Fish type	Survival rate of fries (%)	Survival rate of adults (%)	The average body weight in December (g)
RCC	80.1 ± 4.5	85.7 ± 4.1	260 ± 69
G_1_	82.1 ± 3.8	87.2 ± 5.6	480 ± 55
G_2_	83.5 ± 4.2	86.3 ± 4.2	482 ± 74

## Discussion

In fish, sterile triploid individuals potentially minimize risks associated with accidental releases and are also expected to exhibit higher feeding conversion ratios (FCR) and, consequently, faster growth rates (Foresti et al. 1996). Distant hybridization can result in genome-level alterations including the occurrence of allotriploids and allotetraploids, e.g., F_1_ hybrids of *Ctenopharyngodon idella* × *Hypophthalmichthys nobilis* (Marian and Kraszai 1978) and F_1_ hybrids of *C. auratus* red var. (♀) × *M. amblycephala* (♂) (Liu et al. 2007). The allopolyploids contain both parental genomes, which undergo bivalent pairing at meiosis because only the homologous chromosomes pair (Soltis and Soltis 2000; Wu et al. 2001). Importantly, a diploid-like pairing system prevents meiotic irregularities and improves the efficiency of gamete production in allopolyploid species (Sybenga 1996). However, several studies have documented that many polyploid or diploid individuals of the hybrid progeny of plants show abnormal chromosome behavior during mitosis and meiosis and product gametes with a complete set of paternal or maternal chromosomes (Kasha and Kao 1970; Li et al. 1998; Riera-Lizarazu et al. 2000; Li and Ge 2007). In the present study, following activation by UV-irradiated BSB sperm, the fertilized eggs of 4nF1 developed into normal live gynogenetic offspring without chromosome doubling treatment. Some of these gynogenetic offspring were autotriploids with three sets of chromosomes derived from RCC (3*n* = 150). This indicated that 4nF1 can generated triploid homogametes (3*n* = 150) with three sets of RCC-derived chromosomes. We speculate that the generative cells undergone genomic doubling by premeiotic endoreduplication, endomitosis, or fusion of oogonial germ cells in 4nF1 (Liu et al. 2007). Some showed complete separation of the parental genomes during meiosis, which then developed into gametes with one or more RCC-derived chromosome sets. Indeed, besides autotriploid gamete (3*n* = 150, AAA), haploid (*n* = 50, A), autodiploid (2*n* = 100, AA) and allotetraploids gamete (4*n* = 148, AABB) were also found in 4nF1 (Qin et al. 2014a), and that we have artificially established an autotetraploid fish line (F_2_–F_8_) by fertilization of the autodiploidy diploid eggs and diploid sperm from the females and males of 4nF1 (Qin et al. 2014b)

Autopolyploids generally exhibit random (non-preferential) pairing of chromosomes. Each chromosome has more than one potential partner, which may result in the formation of multivalency during meiosis, and sterility. In nature, gibel carp (*C. auratus gibelio*) is a triploid subspecies of diploid crucian carp, which is able to produce unreduced triploid eggs at the age of 1 year (Vrijenhoek 1994; Gui 1989). The reproductive characteristics of G_1_ are similar to those of gibel carp. G_1_ is autotriploid and can also produce unreduced (3n) eggs at the age of 1 year. Thus, following activation by UV-irradiated BSB sperm and without chromosome doubling treatment, the fertilized eggs of G_1_ are able to develop into a second generation of autotriploid gynogenetic offspring (3*n* = 150, G_2_). We speculate that BSB genetic material has been removed in the autotriploid (G_1_ and G_2_), that can effectively reduced incompatibility and improve autotriploid fertility. Thus, autotriploid reached sexual maturity at 1 year of age, earlier than 4nF1 (sexual maturity at 2 year of age) (Liu et al. 2007). Indeed, gibel carp have the dual reproductive modes of sexual reproduction and gynogenesis, which has been demonstrated using heterologous sperm from other fish species to activate egg and embryo development (Zhou et al. 2000). Our data do not conclusively demonstrate that heterologous sperm (e.g., common carp) activates egg and embryo development of G_1_ as in gibel carp (gynogenesis or hybridogenesis). This is an important aspect and should be investigated in the future.

In many species of cultured fish, females exhibit higher growth rates than males and attain larger sizes (Luo et al. 2011). In addition, the distant crossing or gynogenetic progeny has advantages in growth rates and resistibility. The all-female triploids (G_1_ and G_2_) were obtained by distant hybridization and gynogenesis, which also exhibited higher growth rates than RCC over the entire period. Therefore, these autotriploid provide an important source of gametes for the production of all-female triploids and for the establishment of autotriploid gynogenetic lines. Although autotriploid fish possess three sets of the RCC-derived chromosomes, phenotypic changes obviously occurred. There were clear differences in morphological traits between G_1_ and RCC. The body color of RCC is red while G_1_ are green-brown. Additionally, the ratios of the measurable traits WL/BL, BL/BW, BL/HL, and TL/TW and the countable traits, including the number of lateral line scales, abdominal fins rays, and anal fins rays also differs significantly between G_1_ and RCC, suggesting that this phenotypic variability resulted from genome duplication in the autotriploid fish. Importantly, genomic variation could quickly augment the divergence between homologous chromosomes and then prevent the formation of multivalency during meiosis in G_1_. An interesting question for future study is the mechanism by which unreduced eggs are produced in the autotriploid gynogenetic offspring.
